# Roles and mechanisms of NSUN2-mediated RNA m^5^C modification in cancer progression and immune modulation

**DOI:** 10.3389/fimmu.2025.1702436

**Published:** 2025-11-03

**Authors:** Chunhong Li, Yixiao Yuan, Xiulin Jiang, Qiang Wang

**Affiliations:** ^1^ Department of Oncology, Suining Central Hospital, Suining, Sichuan, China; ^2^ Department of Systems Biology, City of Hope Comprehensive Cancer Center Biomedical Research Center, Monrovia, CA, United States; ^3^ Department of Gastrointestinal Surgical Unit, Suining Central Hospital, Suining, Sichuan, China

**Keywords:** NSUN2, 5-methylcytosine (m5C), RNA epigenetic modification, cancer progression, tumor immune regulation, tumor microenvironment, immune evasion

## Abstract

RNA epigenetic modifications critically regulate gene expression, with 5-methylcytosine (m^5^C) emerging as an important mark in cancer biology. NSUN2, a key m^5^C methyltransferase, modifies diverse RNA species, thereby influencing RNA stability, processing, export, and translation. Accumulating evidence indicates that NSUN2 promotes tumorigenesis by enhancing cell proliferation, supporting drug resistance, driving epithelial–mesenchymal transition, and reprogramming metabolic pathways. Clinically, its dysregulated expression is associated with poor prognosis and potential as a biomarker or therapeutic target. Beyond intrinsic tumor functions, NSUN2 also shapes the tumor immune microenvironment by regulating immune checkpoint molecules, cytokine networks, and immune cell activities, ultimately contributing to immune evasion and influencing immunotherapy efficacy. This review summarizes current insights into the roles and mechanisms of NSUN2 in cancer progression and immune modulation, and discusses challenges and future opportunities for therapeutic exploration.

## 1 Introduction

RNA epigenetic modifications refer to mechanisms by which RNA molecules undergo chemical modifications post-transcriptionally, thereby regulating their structure, function, and fate (1–3). These modifications represent a crucial layer of gene expression regulation. In recent years, with the advancement of high-throughput sequencing technologies, RNA modifications have been found to be widely distributed across various RNA types, including mRNA, tRNA, rRNA, lncRNA, and circRNA, and are involved in regulating RNA stability, splicing, nuclear export, translation efficiency, and cellular stress responses (4–6). Aberrant RNA epigenetic modifications are closely associated with the pathogenesis of various diseases, particularly playing key roles in tumor initiation, progression, and therapy resistance (7–11).

5-Methylcytidine (m^5^C) is an important form of RNA chemical modification, primarily catalyzed by methyltransferases of the NSUN (NOP2/Sun RNA methyltransferase) family (12–15). m^5^C modification can influence RNA stability and translation efficiency, thereby modulating the fine-tuned regulatory networks of gene expression (16–19). In tumor cells, m^5^C modifications have been shown to correlate closely with oncogene expression, cell proliferation, apoptosis evasion, metabolic reprogramming, and invasion and metastasis, suggesting their critical role in tumorigenesis and progression (20–23). m^5^C is a prevalent and evolutionarily conserved RNA modification found in various RNA species, including mRNA, tRNA, rRNA, and non-coding RNAs (24). The distribution of m^5^C sites across transcripts is not random; they are often enriched in the coding sequence and 3′ untranslated regions, where they participate in regulating RNA stability, export, and translation efficiency (21). Functionally, m^5^C plays crucial roles in diverse biological processes such as gene expression regulation, stress response, and cell differentiation. Several m^5^C “reader” proteins have been identified to recognize and interpret this modification, including Y-box binding proteins YBX1 and YBX2, which stabilize m^5^C-modified mRNAs (25); ALYREF, which facilitates the nuclear export of m^5^C-marked transcripts (26); and SRSF2, which contributes to alternative splicing regulation (27). Together, these findings highlight the multifaceted roles of m^5^C in RNA metabolism and underscore the importance of its key methyltransferase NSUN2 in coordinating post-transcriptional gene regulation.

The NSUN family represents the core RNA m^5^C methyltransferases and includes multiple homologous proteins from NSUN1 to NSUN7, among which NSUN2 is one of the most extensively studied members (18, 28–30). NSUN2 mediates m^5^C modifications on tRNA, mRNA, lncRNA, and circRNA, and is involved in regulating RNA stability, processing, nuclear export, and translation efficiency (29, 31, 32). In tumors, aberrant NSUN2 expression is closely associated with the occurrence, progression, and prognosis of various cancers (29, 30, 33). Moreover, increasing evidence indicates that NSUN2 may influence tumor immune evasion and the response to immunotherapy by regulating the expression of immune-related molecules, modulating immune cell functions, and shaping the tumor microenvironment (34, 35). Therefore, a comprehensive understanding of NSUN2’s functions and mechanisms in tumor biology and immune regulation is crucial, as it not only elucidates the molecular basis of tumorigenesis but also provides a theoretical foundation for cancer diagnosis, prognostic evaluation, and targeted therapy.

## 2 Molecular functions and mechanisms of NSUN2

NSUN2 (NOP2/Sun RNA methyltransferase family member 2) is a key member of the NSUN family and serves as a pivotal methyltransferase for RNA 5-methylcytidine m^5^C modification (14). The NSUN2 protein contains a highly conserved S-adenosylmethionine (SAM) binding domain and a catalytic core, enabling it to recognize specific RNA sequences and secondary structures (24, 36). NSUN2 catalyzes the transfer of a methyl group to the C5 position of cytosine to form m^5^C, thereby achieving RNA chemical modification (21, 36). Its catalytic mechanism primarily involves SAM as a methyl donor, with NSUN2 forming an intermediate complex to complete the methyl transfer, accompanied by protein conformational changes to ensure high specificity and catalytic efficiency (37).

**Figure 1:**
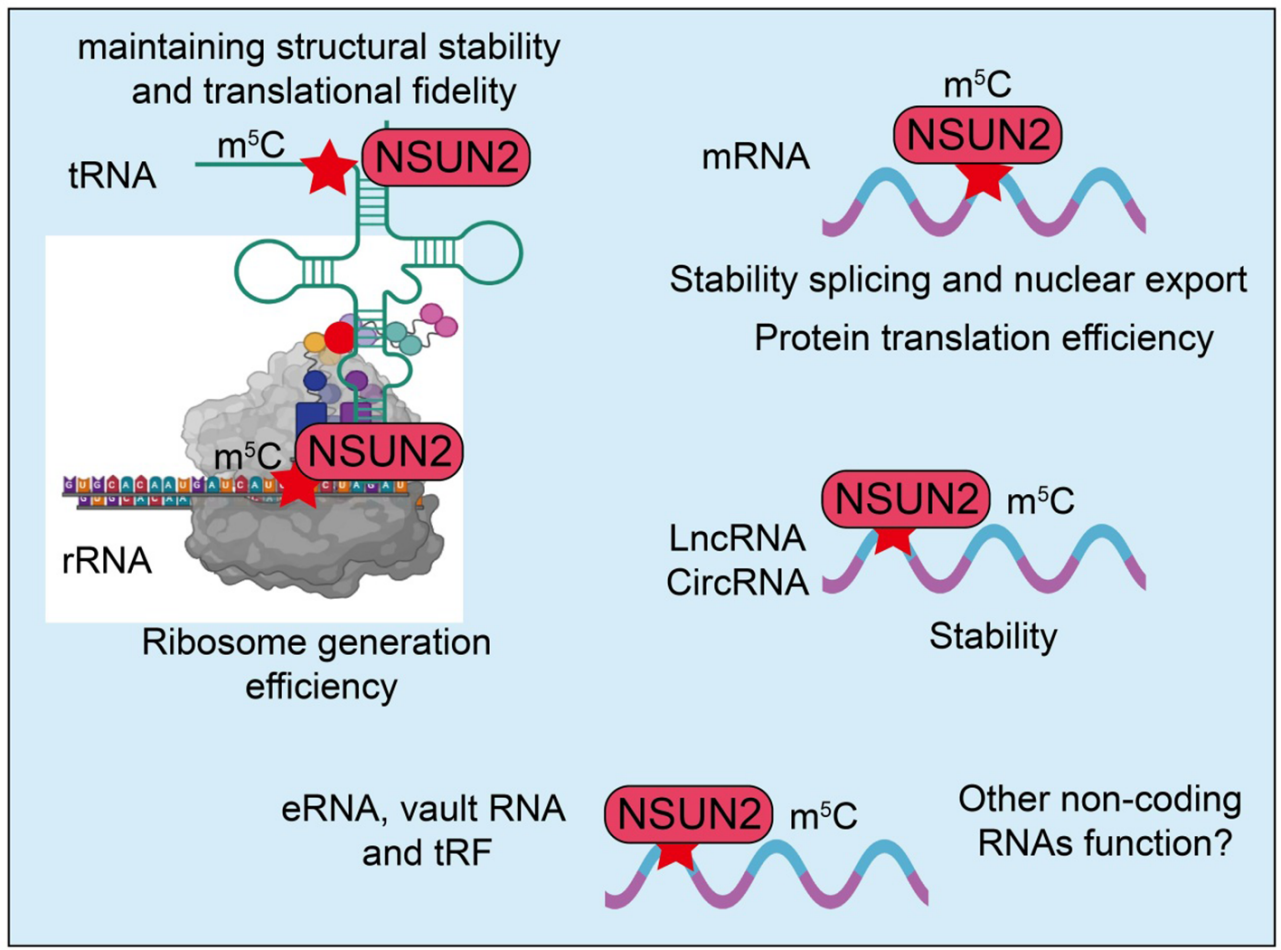
Regulatory roles of NSUN2-mediated m^5^C modification across different RNA types. NSUN2 exerts predominantly positive regulatory effects on RNA metabolism. In tRNAs, NSUN2-catalyzed m^5^C modification maintains structural stability and promotes translational fidelity. In rRNAs, NSUN2 enhances ribosome biogenesis and translational efficiency. For mRNAs, NSUN2-mediated methylation increases RNA stability, facilitates splicing and nuclear export, and promotes protein translation. Similarly, NSUN2-induced m^5^C modification on long non-coding RNAs (lncRNAs) and circular RNAs (circRNAs) enhances transcript stability. NSUN2 may also positively regulate the function and stability of other non-coding RNAs, such as enhancer RNAs (eRNAs), vault RNAs, and tRNA-derived fragments (tRFs). Collectively, NSUN2 acts as a positive post-transcriptional regulator that promotes RNA stability and translation across diverse RNA species.

NSUN2-mediated m^5^C modification targets a wide range of RNAs, including tRNA, rRNA, mRNA, lncRNA, and circRNA (38, 39). m^5^C modification of tRNA contributes to structural stability and translational fidelity while preventing tRNA degradation (40, 41). In mRNA, NSUN2-mediated m^5^C regulates RNA stability (42), splicing (43), and nuclear export (36), thereby affecting translation efficiency (**Figure 1**) (44). For lncRNA and circRNA, m^5^C modifications can alter their structures, protein-binding capacities, and downstream gene regulatory functions, participating in complex cellular signaling networks (45, 46). NSUN2 plays a central role in maintaining RNA homeostasis through m^5^C modification (33, 47). m^5^C can protect RNA from nuclease-mediated degradation, enhancing RNA stability. Furthermore, NSUN2 is critically involved in translation regulation, particularly under stress conditions such as oxidative stress or heat shock (48), where NSUN2-modified RNAs enhance the efficiency and selectivity of protein synthesis, ensuring cellular adaptation to environmental changes (49, 50). Under both homeostatic and stress conditions, NSUN2 not only maintains normal RNA metabolism but also participates in regulating the cell cycle and DNA damage repair (51). Through these functions, NSUN2 plays a pivotal role in cell growth, survival, and environmental adaptation, laying a molecular foundation for its involvement in tumor development and immune regulation.

Recent studies reveal that NSUN2-mediated RNA m^5^C plays a pivotal role in chromatin regulation and leukemia progression (52). In TET2-deficient cells, loss of TET2 promotes chromatin opening via reduced RNA m^5^C oxidation, allowing MBD6 to recognize m^5^C on retrotransposon RNA and remove H2AK119ub, which enhances transcription of self-renewal genes. This pathway is essential for TET2-mutant leukemia survival, and MBD6 inhibition selectively impairs these cells, suggesting a therapeutic target (52). Separately, SRSF2 was identified as an m^5^C reader; its interaction with m5C is disrupted by the leukemia-associated SRSF2^P95H^ mutation. NSUN2 knockdown decreases m^5^C, disrupts SRSF2 binding, alters RNA splicing, and, when combined with SRSF2^P95H^, predicts poor clinical outcomes (27). These findings establish m^5^C as a critical epitranscriptomic bridge linking chromatin dynamics, RNA processing, and oncogenic programs in leukemia. They underscore the therapeutic potential of targeting m^5^C readers (MBD6, SRSF2) or writers (NSUN2) in mutant TET2/SRSF2-driven malignancies, highlighting a convergence of chromatin and RNA-level regulation in leukemogenesis. Future studies could explore combinatorial strategies targeting both RNA modifications and chromatin modulators to selectively eradicate leukemic stem cells while sparing normal hematopoiesis.

## 3 Role of NSUN2 in tumor progression

Across multiple tumor types, NSUN2 acts as a positive regulator of tumor progression by stabilizing target RNAs through m^5^C methylation, enhancing RNA stability, translation efficiency, and splicing, and activating oncogenic signaling pathways (e.g., Ras/PI3K–AKT/ERK, β-catenin, STAT3). Its activity contributes to cell proliferation, metastasis, metabolic reprogramming, and therapy resistance, making NSUN2 a promising prognostic biomarker and therapeutic target. To provide a clearer overview of the functional outcomes of NSUN2-mediated m^5^C modification, **Table 1** summarizes its positive regulatory effects on various RNA species, including enhanced RNA stability, splicing, and translation efficiency.

**Table 1:** Roles and mechanisms of NSUN2 in tumor progression.

Tumor type	Expression	Major biological process	Key downstream targets/pathways	Mechanism of NSUN2 action	Functional outcome	Ref
HCC	Up	Proliferation, metabolism, drug resistance	GRB2, RNF115, AATF, PKM2, MALAT1, GDF15	m^5^C-mediated stabilization of oncogenic mRNAs; activation of Ras and PI3K/AKT signaling; enhancement of glycolysis; ferroptosis suppression	Promotes proliferation, metabolic reprogramming, sorafenib resistance, and poor prognosis	(45, 55, 58, 70)
GC	Up	Proliferation, stemness, glycolysis, drug resistance	PGK1, GCLC, ATG9A, YBX1	Stabilizes PGK1 mRNA via m^5^C/YBX1; enhances PI3K/AKT signaling; lactylation-activated NSUN2 stabilizes GCLC; NSUN2/YBX1 axis promotes autophagy	Promotes growth, invasion, metabolic adaptation, and 5-FU/doxorubicin resistance	(56, 66, 68)
CRC	Up	Proliferation, metastasis	ErbB-STAT3, KSR1 (via LINC02167/YBX1/ILF3)	m^5^C-dependent and -independent mechanisms; stabilizes oncogenic mRNAs and activates ERK/MAPK signaling	Promotes proliferation, invasion, metastasis, and poor prognosis	(57, 60)
PC	Up	Invasion, EMT, metastasis	TIAM2 (YBX1-dependent)	Stabilizes TIAM2 mRNA through m^5^C modification and transcriptional upregulation	Promotes proliferation, migration, and EMT	(61)
OSCC	Up	Metastasis, EMT	UBE2S/β-catenin axis	Stabilizes UBE2S mRNA via m^5^C	Promotes metastasis and EMT	
HNSC	Up	Invasion, metastasis	LAMC2	m^5^C-mediated stabilization of LAMC2 mRNA	Promotes proliferation, invasion, and lymph node metastasis	(62, 63)
BLCA	Up	Drug resistance, proliferation	PRDM11 (via EZH2), R-loop stabilization	Maintains R-loop stability and recruits EZH2 for PRDM11 silencing	Enhances proliferation and cisplatin resistance	(54)
Anaplastic thyroid cancer (ATC)	Up	Drug resistance	SRSF6, AGX2, ABC transporters	NSUN2–ALYREF complex mediates splicing reprogramming; increases glycosylation of ABC transporters	Promotes chemoresistance	(67)
BRAC	Up	Metabolic reprogramming, drug resistance	tRNA^Val-CAC → ALDH3A2, HK1, PFKM	tRNA m^5^C enhances codon-dependent translation of glycolytic enzymes	Promotes glycolysis, proliferation, and paclitaxel resistance	(50, 74)
LC	Up	Metabolic reprogramming	ME1, GLUT3, CDK2	Stabilizes metabolic enzyme mRNAs via m^5^C; regulated by EP300 (H3K27ac)	Promotes metabolism, proliferation, and angiogenesis	(50)
PRAD	Up	Drug resistance	TRIM28	Stabilizes TRIM28 mRNA via m^5^C; upregulated by FOXA1	Promotes proliferation and resistance	(50, 58, 63, 66, 67, 69, 91).
AML	Up	Metabolism, ferroptosis resistance	FSP1, PHGDH, SHMT2	Stabilizes metabolic and ferroptosis-related mRNAs via m^5^C/YBX1	Enhances proliferation, anti-ferroptosis, and survival	(77, 78).
HCC	Up	Lipid metabolism	ACSL6	Stabilizes ACSL6 mRNA via m^5^C	Promotes lipid synthesis and metabolic disorders	(76)

### 3.1 NSUN2 in tumor initiation and proliferation

**Figure 2:**
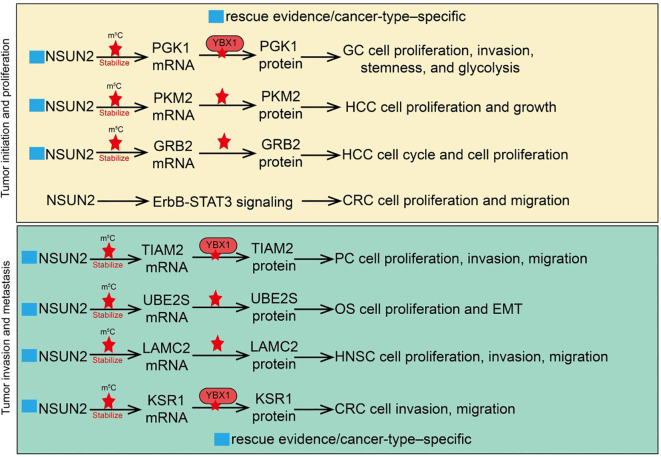
Roles and mechanisms of RNA m5C modification mediated by NSUN2 in tumor progression and metastasis. The blue box indicates that the article includes rescue experiments showing that restoration of certain NSUN2 target genes can only partially rescue the phenotypes caused by NSUN2 loss, and that such effects are restricted to specific cancer types. However, this was not explicitly labeled in the manuscript, which may give the impression that no rescue experiments were performed.

NSUN2 plays a critical role in tumor initiation and proliferation, primarily through gene expression regulation mediated by RNA m^5^C modification (24, 43). Specifically, NSUN2 stabilizes the mRNAs of various oncogenes via m^5^C modification, thereby enhancing their post-transcriptional expression and promoting tumor cell proliferation. For instance, NSUN2 can modify key genes involved in cell cycle regulation, increasing the stability of their mRNAs and facilitating the G1/S phase transition, thereby driving rapid cell proliferation (33, 53). In addition, NSUN2 is functionally important in DNA damage repair. Given the high genomic instability typically observed in tumor cells, NSUN2 enhances the tolerance of tumor cells to DNA damage by regulating the expression of repair-related genes, ensuring their sustained proliferative capacity (51, 54). This mechanism not only maintains tumor cell survival but may also contribute to therapy resistance. For example, in hepatocellular carcinoma (HCC), studies have shown that the overall m^5^C modification level is significantly higher in tumor tissues compared to adjacent normal tissues, and NSUN2 is highly expressed in HCC. Transcriptome analyses further revealed that multiple hyper-methylated m^5^C genes are mainly involved in kinase signaling pathways such as Ras and PI3K-Akt, including GRB2, RNF115, AATF, ADAM15, RTN3, and HDGF (55). Experimental data confirmed that NSUN2 knockdown significantly reduced the mRNA expression of GRB2, RNF115, and AATF, leading to cell cycle arrest in HCC cells. Collectively, NSUN2 regulates the expression of these downstream targets through m^5^C modification, thereby influencing Ras signaling pathway activity (55). Similarly, in gastric cancer (GC), NSUN2 is significantly upregulated in both GC tissues and cell lines. Functional assays demonstrated that NSUN2 silencing inhibits GC cell proliferation, invasion, stemness maintenance, and glycolytic capacity. Mechanistically, NSUN2 stabilizes PGK1 mRNA through m^5^C modification, which is recognized by the m^5^C -binding protein YBX1, thereby enhancing PGK1 expression (56). Notably, PGK1 overexpression can reverse the inhibitory effects of NSUN2 knockdown on GC cell growth, invasion, stemness, and glycolysis (56). Furthermore, the NSUN2/PGK1 axis activates the PI3K/AKT signaling pathway, further promoting tumor progression. In colorectal cancer (CRC), NSUN2 is also markedly upregulated in tumor tissues and is associated with poor patient prognosis. Functional studies indicate that NSUN2 promotes CRC cell proliferation and metastatic potential. Mechanistic investigations revealed that NSUN2 can exert oncogenic effects via non- m^5^C-dependent mechanisms: both wild-type and catalytically inactive NSUN2 mutants upregulate and activate the ErbB-STAT3 signaling pathway in a manner dependent on interaction with CUL4B, whereas CUL4B silencing effectively blocks the non- m^5^C oncogenic function of NSUN2 (57). Additionally, in HCC, NSUN2 is significantly overexpressed and correlates with poor postoperative prognosis. NSUN2 overexpression markedly promotes HCC cell proliferation and metastasis, while NSUN2 knockdown inhibits tumor growth and invasion (58). Importantly, NSUN2-mediated m^5^C hypermethylation is closely associated with elevated mRNA expression and contributes to metabolic reprogramming in HCC. Mechanistically, the glycolytic terminal enzyme PKM2 has been identified as a key downstream target of NSUN2-mediated m^5^C modification (58). NSUN2 enhances PKM2 mRNA stability by increasing m^5^C levels at the C773 site of its 3’-UTR, thereby promoting tumor metabolism and proliferation. In summary, NSUN2 exerts a central role in tumor cell proliferation, metastasis, metabolic reprogramming, and therapy resistance through both m^5^C-dependent and -independent mechanisms, functioning as a critical oncogenic factor in multiple cancers and representing a potential therapeutic target (**Figure 2** **) (** 58).

### 3.2 NSUN2 in tumor invasion and metastasis

Tumor invasion and metastasis are hallmarks of malignant progression and are major contributors to the high mortality of cancer (59). NSUN2 plays a critical regulatory role in these processes, primarily through RNA m^5^C modification, which affects the expression and function of relevant genes (60). In pancreatic cancer (PC), NSUN2 is significantly upregulated in tumor tissues and is associated with invasive clinical features (61). NSUN2 silencing inhibits PC cell proliferation, migration, and invasion, while reducing tumor growth and metastasis *in vivo*. Conversely, NSUN2 overexpression promotes tumor growth and metastasis. Mechanistic studies reveal that NSUN2 stabilizes TIAM2 mRNA via m^5^C modification and delays its degradation in a YBX1-dependent manner, while partially enhancing TIAM2 transcription to exert oncogenic effects (61). Disruption of the NSUN2/TIAM2 axis suppresses epithelial-mesenchymal transition (EMT), thereby attenuating the malignant phenotype of PC cells (61). The critical role and molecular mechanisms of NSUN2 in osteosarcoma progression and metastasis have also been elucidated. NSUN2 is markedly upregulated in osteosarcoma tissues and cell lines and correlates with poor patient prognosis. NSUN2 significantly promotes osteosarcoma cell metastasis and EMT. Mechanistically, NSUN2 stabilizes UBE2S mRNA through m^5^C modification, thereby enhancing the activity of the UBE2S/β-catenin axis (62), which promotes cell invasion and EMT. UBE2S overexpression can rescue the inhibitory effects on invasion and EMT induced by NSUN2 knockdown. In head and neck squamous cell carcinoma (HNSCC) (62), NSUN2 is significantly upregulated in tumor tissues and is associated with poor prognosis (63). Functional assays demonstrate that NSUN2 knockdown inhibits cancer cell proliferation, migration, and invasion, and reduces tumor formation and lymph node metastasis *in vivo (* 63). NSUN2 knockdown alters the genome-wide m^5^C modification pattern, with LAMC2 identified as a key downstream target (63). NSUN2 regulates LAMC2 mRNA stability and expression via m^5^C modification, thereby promoting malignant phenotypes and metastatic potential of HNSCC cells (63). In colorectal cancer (CRC), the long non-coding RNA LINC02167 is markedly upregulated in tumor tissues and closely associated with advanced clinical features and poor prognosis (60). LINC02167 promotes CRC cell migration and invasion and enhances metastatic capacity *in vivo*. Mechanistic studies show that LINC02167 functions as a molecular scaffold, forming a complex with YBX1 and ILF3, which facilitates YBX1 recognition and binding of NSUN2-mediated m^5^C modification sites on KSR1 mRNA, thereby stabilizing KSR1 mRNA and activating the ERK/MAPK signaling pathway to drive CRC metastasis (60). Additionally, MYC-driven transcriptional activation upregulates LINC02167 expression, further reinforcing this pro-metastatic signaling axis (60). In summary, NSUN2 exerts a central role in invasion and metastasis across multiple tumor types through m^5^C modification and associated downstream gene axes. These studies not only reveal the key molecular mechanisms by which NSUN2 drives malignant progression but also provide a theoretical basis for developing anti-metastatic therapeutic strategies targeting NSUN2 and its downstream effectors (**Figure 2**).

### 3.3 NSUN2 in tumor drug resistance

**Figure 3:**
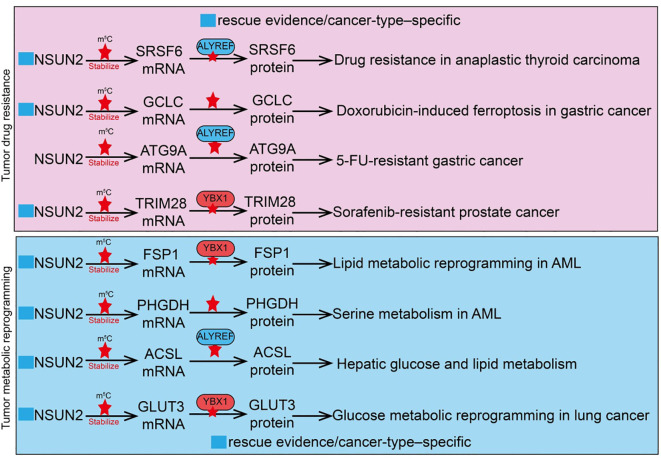
Roles and mechanisms of RNA m5C modification mediated by NSUN2 in tumor drug resistance and metabolic reprogramming. The blue box indicates that the article includes rescue experiments showing that restoration of certain NSUN2 target genes can only partially rescue the phenotypes caused by NSUN2 loss, and that such effects are restricted to specific cancer types. However, this was not explicitly labeled in the manuscript, which may give the impression that no rescue experiments were performed.

Tumor drug resistance is a major cause of treatment failure and cancer relapse, involving multiple molecular mechanisms (21, 64). These primarily include overexpression of drug efflux pumps, enhanced DNA damage repair pathways, inhibition of apoptotic signaling, and protective effects of the tumor microenvironment (64). RNA epigenetic modifications also play crucial roles in the development of resistance, for instance, by regulating mRNA stability, translation efficiency, or non-coding RNA functions of key genes, thereby affecting drug target expression and response (21, 65, 66). As an m^5^C methyltransferase, NSUN2 can modify oncogenes or DNA repair-related gene RNAs to enhance tumor cell tolerance to chemotherapeutic agents such as cisplatin or paclitaxel (51). NSUN2 also regulates immune-related pathways, further promoting drug resistance and immune evasion, indicating that targeting NSUN2 and its downstream pathways may provide novel strategies to overcome tumor resistance. For example, in bladder cancer (BCa), R-loop levels are significantly elevated, and NSUN2 binds and stabilizes R-loop structures through its m^5^C methyltransferase activity (54). NSUN2 recruits the histone methyltransferase EZH2 to epigenetically silence the tumor suppressor PRDM11, thereby promoting BCa cell proliferation and tumor progression. Functional assays show that NSUN2 knockdown enhances tumor sensitivity to cisplatin, increases DNA damage levels, and impairs homologous recombination repair due to reduced MRE11 recruitment (54). In anaplastic thyroid cancer (ATC), high NSUN2 expression is strongly associated with drug resistance. As an m^5^C “writer,” NSUN2 cooperates with the m^5^C “reader” ALYREF on SRSF6 mRNA to induce selective splicing reprogramming, switching the UAP1 isoform from AGX1 to AGX2 (67). AGX2 enhances N-glycosylation of ABC transporters, stabilizing their protein levels and preventing ubiquitin-mediated degradation, thus promoting a drug-resistant phenotype. NSUN2 inhibitors have been shown to suppress its enzymatic activity and reduce downstream target expression, providing a potential therapeutic strategy to overcome ATC resistance (67). In gastric cancer (GC), NSUN2 mediates tumor cell survival in acidic microenvironments through lactate-induced lysine lactylation. Lactylation at lysine 508 (K508) enhances NSUN2 methyltransferase activity, enabling it to target the mRNA of glutamate-cysteine ligase catalytic subunit (GCLC), promoting m^5^C modification and stabilizing GCLC expression (66). Activated GCLC increases intracellular glutathione (GSH) levels and reduces lipid peroxidation, conferring resistance to doxorubicin-induced ferroptosis (66). Introduction of NSUN2 K508R or GCLC C-A (mutation of five cytosine sites) almost abolishes this pathway. N-α-acetyltransferase 10 (NAA10) is identified as the lactylase of NSUN2, and lactate treatment significantly enhances the interaction between NSUN2 and NAA10, thereby activating NSUN2 (66). Similarly, in 5-fluorouracil (5-FU) resistant GC, the m^5^C reader YBX1 is significantly upregulated in resistant cell lines and patient tissues, promoting autophagy and the resistant phenotype (68). Mechanistically, the transcription factor MAZ binds to the YBX1 promoter to drive its transcriptional upregulation; YBX1 then stabilizes ATG9A mRNA through NSUN2-mediated m^5^C modification, enhancing autophagic activity and facilitating 5-FU resistance. Functional experiments and clinical data indicate that high YBX1 expression is closely associated with poor prognosis in advanced GC patients receiving 5-FU chemotherapy (68). In sorafenib-resistant hepatocellular carcinoma (HCC), NSUN2 cooperates with ALYREF to maintain the stability and high expression of the long non-coding RNA MALAT1 via its m^5^C methyltransferase activity (45). In resistant cells, the NSUN2/ALYREF/MALAT1 axis is activated, and MALAT1 stabilizes SLC7A11 mRNA by directly binding to ELAVL1 and promoting its cytoplasmic translocation, thereby inhibiting sorafenib-induced ferroptosis and promoting the resistant phenotype. Functional studies further show that combining the MALAT1 inhibitor MALAT1-IN1 with sorafenib significantly enhances drug efficacy (45). In prostate cancer, NSUN2 adds m^5^C modifications to TRIM28 mRNA, increasing its stability and promoting TRIM28 protein expression (69). The prostate cancer-specific transcription factor FOXA1 transcriptionally activates NSUN2 expression. Similarly, in hypoxia-mediated sorafenib-resistant HCC, hypoxia significantly reduces HCC cell sensitivity to sorafenib, as evidenced by increased IC50 and decreased apoptosis. Mechanistic studies indicate that hypoxia-induced HIF1A upregulates NSUN2, which stabilizes GDF15 mRNA via m^5^C modification, leading to increased GDF15 secretion (70). Neutralization of GDF15 inhibits the Akt/mTOR signaling pathway, enhancing HCC cell sensitivity to sorafenib (70). In summary, NSUN2 plays a central role in chemotherapeutic and targeted therapy resistance across multiple tumor types through diverse m^5^C-dependent and microenvironment-mediated mechanisms. These studies reveal the key molecular mechanisms of NSUN2 in drug resistance and highlight NSUN2 and its downstream pathways as potential therapeutic targets to overcome tumor resistance (**Figure 3**).

### 3.4 NSUN2 in tumor metabolic reprogramming

Tumor metabolic reprogramming is a key biological feature that enables cancer cells to adapt to rapid proliferation and malignant progression (71). At its core, this process involves remodeling metabolic pathways to meet energy, precursor, and antioxidant demands (71). Cancer cells often exhibit enhanced glycolysis (Warburg effect), lipid synthesis, and amino acid metabolism, accompanied by selective regulation of mitochondrial oxidative phosphorylation. These metabolic alterations not only support rapid cell proliferation but also provide stress resistance and chemotherapy tolerance (72). Recent studies indicate that RNA epigenetic modifications, particularly NSUN2-mediated m^5^C modification of tRNAs or mRNAs, can directly regulate the translation or stability of key metabolic enzymes, thereby promoting glycolysis, lipid metabolism, and glutathione synthesis, which in turn drives tumor metabolic reprogramming (73). Metabolic reprogramming not only affects tumor cell growth and survival but also reshapes the tumor microenvironment, influencing immune cell infiltration and antitumor immune responses, providing potential avenues for combined metabolic and epigenetic-targeted therapies. In triple-negative breast cancer (TNBC), NSUN2 is significantly upregulated and correlates with poor prognosis. Mechanistic studies demonstrate that NSUN2 mediates m^5^C modification of tRNA^Val^-CAC, enhancing codon-dependent translation of key glycolytic genes, including ALDH3A2, ALDH7A1, HK1, and PFKM (74). Loss of NSUN2 disrupts tRNA^Val^-CAC m^5^C modification, suppresses translation of these metabolic enzymes and glycolytic activity, thereby inhibiting TNBC cell proliferation, migration, and invasion (74). NSUN2 overexpression enhances paclitaxel resistance, whereas its inhibition increases TNBC sensitivity to paclitaxel. In lung cancer, NSUN2 is significantly upregulated in Cr(VI)-transformed cells and lung tissues from exposed mice. NSUN2 inhibition reduces cell proliferation, migration, clonogenicity, and tube formation. Mechanistically, NSUN2 enhances the stability of ME1, GLUT3, and CDK2 mRNAs through m^5^C modification, promoting metabolic reprogramming and cell cycle progression, thereby conferring proliferative and angiogenic advantages (50). The m^5^C reader protein ALYREF participates in NSUN2-mediated m^5^C functions, and EP300 transcriptionally activates NSUN2 via H3K27ac histone modification, regulating Cr(VI)-induced carcinogenesis (50). In hepatocellular carcinoma (HCC), malignant cells and CD8^+^ T cells exhibit distinct glucose metabolic patterns during tumor evolution. A glucose metabolism advantage drives NSUN2 upregulation in tumor cells, which stabilizes key glycolytic gene transcripts (GLUT1, HK2, PFKM) via mRNA methylation, enhancing tumor cells’ competitive advantage in glucose uptake (75). This forms a positive feedback loop, accelerating malignancy and exacerbating CD8^+^ T cell dysfunction. Based on this mechanism, combined targeting of the GLUT1/NSUN2 axis with the small molecule inhibitor WZB117 and PD-L1 immune checkpoint blockade synergistically suppresses tumor evolution and reverses immune suppression, offering a potential strategy for therapy-resistant HCC (75). NSUN2 is also significantly upregulated in liver tissues from type 2 diabetes mellitus (T2DM) patients and high-fat diet (HFD) mice (76). NSUN2 knockdown improves glucose tolerance and pyruvate metabolism, alleviates insulin resistance, and reduces hepatic lipid accumulation. Mechanistically, NSUN2 stabilizes ACSL6 mRNA via m^5^C modification, promoting lipid synthesis and accumulation (76). NSUN2 deficiency reduces ACSL6 expression and improves metabolic phenotypes, while ACSL6 overexpression partially rescues the metabolic improvements induced by NSUN2 knockdown, confirming the functional role of the NSUN2-ACSL6 axis in abnormal hepatic glucose and lipid metabolism. In acute myeloid leukemia (AML), NSUN2 is aberrantly upregulated in patient samples and correlates with poor prognosis. NSUN2 promotes leukemia cell proliferation, enhances xenograft growth, and confers resistance to ferroptosis (77). Mechanistically, NSUN2 catalyzes m^5^C modification on the 3’UTR of FSP1 (ferroptosis suppressor protein 1) mRNA, allowing recognition and stabilization by the m^5^C reader YBX1, which inhibits lipid peroxidation and oxidative damage, protecting AML cells from ferroptotic stress. NSUN2 or FSP1 knockout induces mitochondrial remodeling and increases ferroptosis sensitivity, whereas wild-type NSUN2 or FSP1 rescues resistance, while catalytically inactive NSUN2 or function-deficient FSP1 cannot (77). Additionally, NSUN2 knockdown inhibits AML cell proliferation and clonogenicity while promoting apoptosis; in mouse AML models, NSUN2 silencing reduces tumor burden and prolongs survival. Mechanistically, NSUN2 stabilizes mRNAs of key enzymes in the serine/glycine biosynthesis pathway, including PHGDH and SHMT2, through m^5^C modification, enhancing metabolic activity and supporting leukemia cell proliferation (78). These findings elucidate the NSUN2-m^5^C-PHGDH/SHMT2 regulatory axis in AML pathogenesis and highlight its potential as a therapeutic target (78). In summary, NSUN2 exerts a central role in tumor metabolic reprogramming via RNA m^5^C modification. It stabilizes mRNAs of key genes involved in glycolysis, lipid metabolism, and amino acid metabolism, enhancing energy supply and stress resistance. Moreover, NSUN2 modulates ferroptosis, glucose metabolic competitiveness, and drug resistance, further promoting tumor proliferation, invasion, and therapy resistance. NSUN2-mediated metabolic regulation also affects the tumor microenvironment and immune cell function, supporting immune evasion. These studies reveal the multidimensional role of NSUN2 in tumor metabolic adaptation and malignant progression, suggesting that targeting NSUN2 and its downstream metabolic axes may provide novel combinatorial therapeutic strategies for various solid tumors and hematologic malignancies (**Figure 3**).

Glucose metabolism plays a central role in tumorigenesis, but whether glucose can directly act as a signaling molecule to regulate oncoprotein activity has remained unclear (79). Recent evidence positions glucose not only as a metabolic substrate but also as a direct signaling molecule that drives tumorigenesis. Glucose binds to the methyltransferase NSUN2, promoting its oligomerization and activation, which maintains global m5C RNA methylation and stabilizes key targets such as TREX2. By sustaining TREX2 expression, NSUN2 limits cytosolic dsDNA accumulation and suppresses cGAS/STING-mediated innate immune responses, thereby facilitating tumor progression and resistance to anti-PD-L1 therapy (80). Disruption of glucose binding or NSUN2 function activates cGAS/STING signaling, enhancing apoptosis and CD8^+^ T cell infiltration, and overcoming immunotherapy resistance. These findings highlight a previously unrecognized glucose/NSUN2/TREX2 axis that links nutrient sensing to epitranscriptomic regulation and immune evasion, suggesting that targeting this pathway could simultaneously inhibit tumor growth and sensitize immunologically “cold” tumors to checkpoint blockade (80). Collectively, these findings establish NSUN2 as a critical epitranscriptomic hub linking tumor metabolism and immune regulation, and suggest that co-targeting NSUN2-mediated m^5^C pathways and metabolic or immune checkpoints may represent a promising strategy for overcoming therapy resistance.

### 3.5 NSUN2 as a prognostic biomarker

Multiple clinical studies have demonstrated that NSUN2 is significantly upregulated across various solid tumors and hematologic malignancies, including pancreatic cancer, acute myeloid leukemia (AML), osteosarcoma, colorectal cancer (CRC), head and neck squamous cell carcinoma (HNSCC), and gastric cancer (39, 53). High NSUN2 expression is generally associated with advanced tumor stage, enhanced tumor invasiveness, increased metastatic risk, and predicts reduced overall survival (OS) and progression-free survival (PFS). Furthermore, NSUN2 expression can be assessed through histological analysis or RNA sequencing, providing valuable information for tumor diagnosis, prognostic evaluation, and therapeutic response prediction (81). Given its multifaceted roles in tumor initiation, proliferation, metastasis, and drug resistance, NSUN2 serves not only as a potential biomarker but also as a candidate target for therapy. Targeting NSUN2 and its downstream signaling axes, such as TIAM2, FSP1, UBE2S, and PHGDH, holds promise for improving treatment responses in cancer patients (24, 29). Such strategies could be combined with chemotherapy, immunotherapy, or molecularly targeted agents to achieve synergistic antitumor effects.

## 4 NSUN2 and tumor immune regulation

### 4.1 Impact of NSUN2 on the immune microenvironment

**Figure 4:**
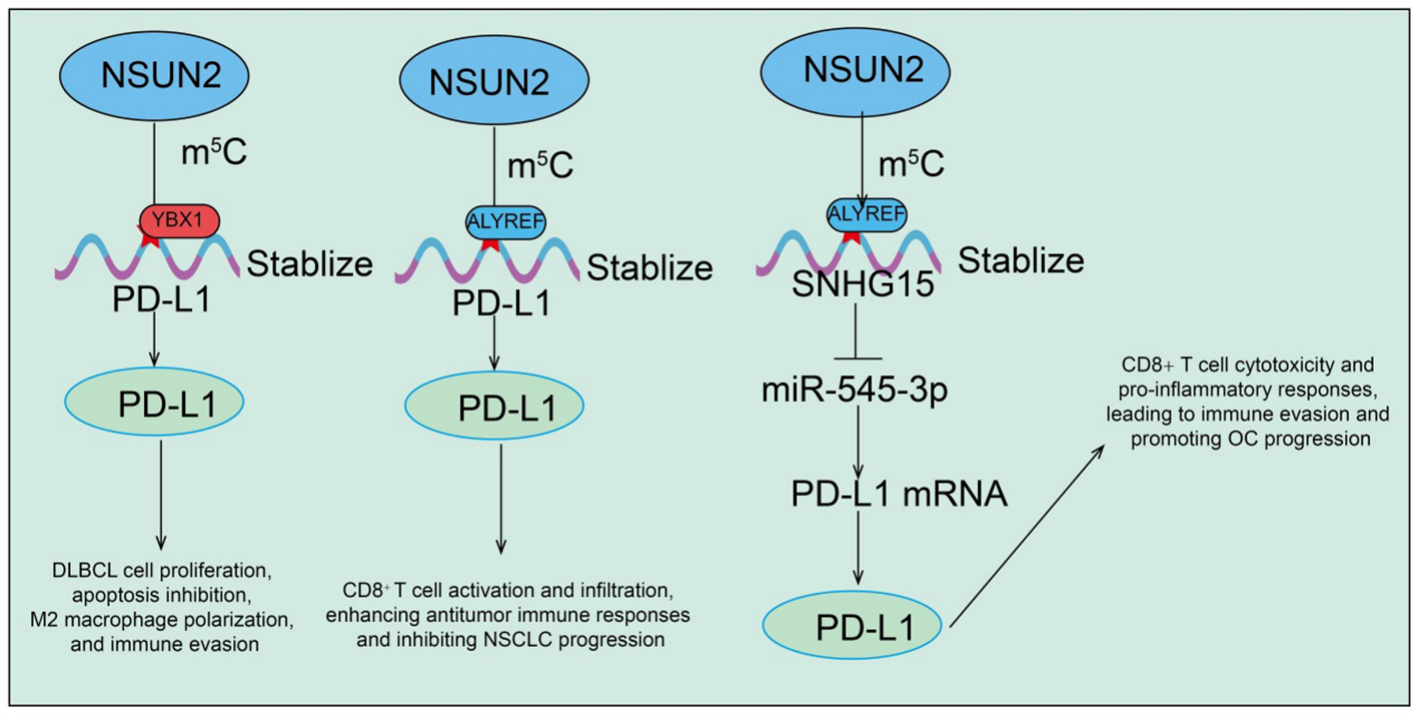
Functions and mechanisms of RNA m5C modification mediated by NSUN2 in regulating PD-L1 expression.

Recent studies increasingly indicate that the tumor immune microenvironment (TIME) plays a critical role in cancer initiation, progression, and therapeutic response. As an RNA m^5^C methyltransferase, NSUN2 not only regulates intrinsic tumor cell proliferation and metabolism but also significantly modulates the immune state of the tumor microenvironment through regulation of immune-related genes and molecules. In diffuse large B-cell lymphoma (DLBCL), NSUN2 is markedly upregulated in both tissues and cells and can be transferred intercellularly via tumor-derived exosomes (34). Exosomal NSUN2 promotes DLBCL cell proliferation, inhibits apoptosis, induces M2 macrophage polarization, and enhances immune evasion (34). Mechanistic studies reveal that NSUN2 stabilizes PD-L1 mRNA expression via m^5^C- and YBX1-dependent pathways (34). Functional experiments show that PD-L1 inhibition significantly diminishes the proliferative, anti-apoptotic, M2-polarizing, and immune-evasive effects of exosomal NSUN2 on DLBCL cells (34). These findings highlight the exosomal NSUN2-m^5^C-YBX1-PD-L1 regulatory axis as a key modulator of tumor progression and immune regulation in DLBCL, providing a potential therapeutic target (34). In non-small cell lung cancer (NSCLC), both NSUN2 and its m^5^C reader protein ALYREF are significantly upregulated and contribute to tumor cell proliferation and progression. Mechanistic analyses identify PD-L1 as a downstream target of NSUN2-mediated m^5^C modification (35). NSUN2 stabilizes PD-L1 mRNA in an ALYREF-dependent manner, and NSUN2 knockdown significantly reduces m^5^C modification on PD-L1 mRNA, decreases its stability, and thereby enhances CD8^+^ T cell activation and infiltration, improving antitumor immune responses (35). In ovarian cancer (OC), the long non-coding RNA SNHG15 is markedly upregulated and regulated by NSUN2-mediated m^5^C modification. Functional studies demonstrate that SNHG15 acts as a molecular sponge for miR-545-3p, relieving suppression of its downstream target PD-L1 (82). This process promotes OC cell proliferation while suppressing CD8^+^ T cell cytotoxicity and pro-inflammatory responses, thereby facilitating immune evasion (**Figure 4** **) (** 82). Collectively, NSUN2 regulates the tumor immune microenvironment and promotes immune escape in various cancers through multiple molecular axes, including PD-L1, YBX1, ALYREF, and the SNHG15/miR-545-3p pathway. These findings suggest that targeting NSUN2 and its downstream m^5^C-related pathways may enhance the efficacy of immunotherapy and provide a rational basis for combination strategies with immune checkpoint inhibitors.

### 4.2 Regulation of immune cell function

Recent studies have demonstrated that RNA epigenetic modifications play critical roles in regulating immune cell function and shaping the tumor immune microenvironment (TIME) (83). As an RNA 5-methylcytosine (m^5^C) methyltransferase, NSUN2/Nsun2 has emerged as an important regulator in various immune-modulatory processes and tumor progression. In mouse CD4^+^ T cells (84), differentiation and alleviates Th17-mediated colitis pathology. Mechanistic studies indicate that the key transcription factor RORγt recruits Nsun2 to the chromatin regions of its target genes, including Il17a and Il17f, where Nsun2 catalyzes m^5^C formation via a transcription-coupled mechanism, thereby enhancing the stability of related mRNAs (84). This demonstrates an intrinsic role of m^5^C modification in regulating immune cell function at the cellular level. In the tumor context, NSUN2 also exhibits significant immunoregulatory functions. In nasopharyngeal carcinoma (NPC), NSUN2 overexpression correlates closely with poor patient prognosis (85). Functional experiments show that NSUN2 promotes NPC cell proliferation, migration, and invasion. Differentially expressed genes between high and low NSUN2 expression patients are enriched in pathways related to activation and proliferation of various immune cells. Further analysis indicates that NSUN2 negatively regulates immune cell infiltration in the NPC tumor microenvironment (TME) (85), suggesting that its expression level may be inversely correlated with sensitivity to immunotherapy and chemotherapy. In clear cell renal cell carcinoma (ccRCC), NSUN2 has been identified as a critical m^5^C methyltransferase with prognostic and therapeutic relevance. Functional studies reveal that NSUN2 stabilizes NEO1 mRNA (86), thereby promoting cell proliferation, migration, and invasion, while also reprogramming tumor glycolytic metabolism and histone lactylation levels. Additionally, NSUN2 upregulates PD-L1 expression in a lactylation-dependent manner via the MYC/POM121/CD274 axis, inducing immune evasion. NSUN2 silencing enhances *in vitro* CD8^+^ T cell cytotoxicity and *in vivo* TNF-α^+^ T cell infiltration (86). Collectively, NSUN2 links immune cell function with tumor glycolytic metabolism and immune evasion. These findings reveal the dual role of NSUN2 in tumor immune regulation and provide potential mechanistic insights and therapeutic targets for tumors including NPC and ccRCC.

### 4.3 Relationship with immunotherapy

Recent studies have demonstrated that NSUN2 significantly influences the tumor immune microenvironment (TME) by regulating the expression of key immune-related genes through its RNA 5-methylcytosine (m^5^C) methyltransferase activity. Specifically, NSUN2 can stabilize the mRNAs of PD-L1, FSP1, and other immunosuppressive factors, enhancing the immunosuppressive capacity of tumor cells, leading to impaired CD8^+^ T cell function and the establishment of an immunosuppressive TME (86). Furthermore, in certain tumor types, NSUN2-mediated m^5^C modification can indirectly modulate T cell infiltration and antitumor immune responses through the regulation of metabolic reprogramming pathways, including glycolysis and lipid metabolism. Notably, tumors with high NSUN2 expression often exhibit upregulated PD-L1 and immunosuppressive TME characteristics, suggesting that NSUN2 may contribute to reduced efficacy of immune checkpoint inhibitors (ICIs, e.g., anti-PD-1/PD-L1) (34). Preclinical and clinical evidence indicates that NSUN2 inhibition can decrease the expression of immunosuppressive factors and enhance CD8^+^ T cell cytotoxicity, implying that NSUN2 status may serve as a novel biomarker to predict ICI responsiveness. Based on these findings, combination strategies hold significant therapeutic potential. In tumors with elevated NSUN2 expression, simultaneous NSUN2 inhibition and ICI therapy may achieve synergistic antitumor effects: NSUN2 inhibition attenuates tumor-derived immunosuppressive signaling, while ICIs restore T cell function, resulting in dual immune activation (35). Future targeting approaches may include small molecule inhibitors, mRNA interference, or CRISPR/Cas9-based strategies, integrated with optimized immunotherapy regimens. By profiling tumor NSUN2 expression and m^5^C modification landscapes, patients suitable for combined NSUN2 inhibition and ICI therapy can be selected, enabling precision immunotherapy. Looking ahead, systematic dissection of NSUN2’s role across different tumor TMEs—particularly its regulation of immune cell infiltration and metabolism—will elucidate its dynamic contribution to tumor-immune co-evolution (86). Concurrently, the development of potent and specific NSUN2 inhibitors and preclinical validation of their safety and efficacy in combination with ICIs will provide novel strategies for precision cancer immunotherapy. Integrating multi-omics and single-cell sequencing approaches, the NSUN2-m^5^C modification landscape may emerge as a critical biomarker for predicting ICI efficacy, thereby facilitating the clinical translation of individualized immunotherapy.

## 5 Advantages and limitations of targeting NSUN2 in cancer therapy

Targeting NSUN2 presents a promising avenue in precision oncology. NSUN2 mediates m^5^C modifications to regulate the expression of key metabolic enzymes and immune-related genes, thereby promoting tumor cell proliferation, migration, stemness maintenance, and immune evasion (87). Therapeutically targeting NSUN2 can directly interfere with these critical molecular pathways, inhibiting tumor growth and metastasis while reducing the expression of immunosuppressive factors, enhancing CD8^+^ T cell activation and infiltration, and providing a potential strategy for combination with immunotherapy (29). Furthermore, NSUN2 upregulation is closely associated with chemotherapeutic resistance in multiple tumor types (24). Its inhibition has been shown to restore drug sensitivity, offering a novel approach to overcome therapy resistance (80). Therefore, NSUN2 not only serves as a potential antitumor target but also holds promise as a biomarker for predicting responses to immunotherapy and chemotherapy. However, targeting NSUN2 also faces several challenges (88). Currently, there is a lack of highly specific inhibitors, and the conserved nature of its catalytic site increases the difficulty of drug development (24). Additionally, NSUN2 performs essential functions in normal cells, including the maintenance of stem cell properties and normal metabolic processes; non-specific inhibition may result in undesirable side effects. Moreover, NSUN2 expression levels vary among different tumor types and individual patients, necessitating biomarker-guided patient stratification and treatment optimization in accordance with the characteristics of the tumor microenvironment.

Recent studies have revealed that the NSUN family of RNA methyltransferases plays critical roles in diverse physiological and pathological processes; however, selective inhibitors with robust cellular activity have long been lacking. A recent study employed cysteine-directed activity-based protein profiling (ABPP) and identified azetidine acrylamides as stereoselective, irreversible covalent inhibitors that specifically target the conserved catalytic cysteine of human NSUN2 (89). Although this site is highly conserved among NSUN family members, these inhibitors exhibited minimal cross-reactivity with other NSUN proteins and demonstrated favorable proteome-wide selectivity (89). Functional assays confirmed that these compounds effectively suppressed the catalytic activity of recombinant NSUN2 without affecting NSUN6, and stereoselectively disrupted the interaction between NSUN2 and tRNA in cancer cells, leading to a global reduction of tRNA m^5^C levels (89). Nevertheless, this work primarily provides a chemical tool compound to demonstrate that NSUN2 can be selectively inhibited by small molecules, while its pharmacokinetic properties, *in vivo* applicability, and therapeutic potential in disease models remain largely unexplored. Therefore, these inhibitors currently serve mainly as research probes for dissecting RNA methylation mechanisms, and their clinical translatability is still limited. Against this backdrop, our study further investigates the disease-related functions of NSUN2 and lays the groundwork for the future development of NSUN2 inhibitors with therapeutic potential. Although this study has reported the development of azetidine acrylamides as stereoselective covalent inhibitors of NSUN2, these compounds currently function primarily as chemical tool molecules rather than therapeutic candidates. Specifically, they were designed to demonstrate the feasibility of selective NSUN2 inhibition, but they have not been evaluated for pharmacokinetic properties, *in vivo* stability, or bioavailability, which are essential for translational applications. Moreover, their inhibitory activity has so far only been validated *in vitro* and in cultured cells, without evidence of efficacy in animal disease models. It also remains unclear whether these inhibitors can effectively modulate NSUN2-dependent regulatory pathways in tumor progression or provide therapeutic benefit in relevant cancer contexts. Therefore, while these inhibitors represent an important step forward for probing NSUN2 biology, they are not yet capable for the implied therapeutic application, which underscores the unmet need for developing clinically viable NSUN2 inhibitors.

Future research should focus on the development of potent and specific NSUN2 inhibitors and explore their synergistic effects in combination with immune checkpoint inhibitors. Stratification of patients based on NSUN2 expression and m^5^C modification profiles may enable precision and individualized therapeutic interventions. Systematic elucidation of NSUN2’s mechanistic roles across diverse tumor types, particularly in tumor metabolism and immune regulation, will provide a solid theoretical foundation for the clinical application of NSUN2-targeted therapies and potentially establish NSUN2 as a key component of next-generation cancer treatment strategies.

## 6 Conclusion and perspectives

NSUN2, as an RNA m^5^C methyltransferase, exerts dual roles in tumor biology and tumor immune regulation. On one hand, NSUN2 promotes tumor cell proliferation, invasion, metastasis, and therapy resistance by regulating RNA stability, translation efficiency, and the expression of metabolism-related genes (90). Several studies demonstrated that restoration of individual NSUN2 target genes, such as PGK1, PKM2, GRB2, TIAM2, UBE2S, LAMC2, KSR1, SRSF6, GCLC, TRIM28, FSP1, PHGDH, ACSL, or GLUT3, could only partially rescue the phenotypes induced by NSUN2 depletion (50, 58, 63, 66, 67, 69, 91). This observation suggests that the oncogenic functions of NSUN2 cannot be attributed to a single downstream effector but rather arise from the concerted regulation of multiple targets. In addition, the rescuing effects of these genes are often observed only in specific tumor types, reflecting the context-dependent nature of NSUN2 activity. For instance, genes involved in glycolysis or redox balance (e.g., PGK1, PKM2, GCLC, PHGDH) are particularly relevant in metabolically reprogrammed tumors, whereas adhesion- and signaling-related targets (e.g., LAMC2, KSR1, TIAM2) play predominant roles in highly invasive cancers. Therefore, NSUN2 should be regarded as a pleiotropic regulator that coordinates diverse, tumor-type–specific pathways, which explains why rescue of a single gene cannot fully recapitulate the oncogenic impact of NSUN2.

On the other hand, NSUN2 contributes to tumor immune evasion and modulates responses to immunotherapy by influencing the expression of immunosuppressive factors, regulating immune cell function, and shaping the tumor microenvironment. Thus, NSUN2 occupies a central molecular position in both tumor progression and immune regulation (33). Despite these advances, several challenges and knowledge gaps remain. The target spectrum of NSUN2 is broad and diverse, and its specific regulatory networks are not yet fully elucidated. Furthermore, its functional roles may differ across tumor types and microenvironmental contexts, necessitating further investigation (29). The mechanistic understanding of NSUN2 in tumor immunity also remains incomplete, highlighting the need for more in-depth mechanistic studies and clinical validation. Single-cell sequencing and multi-omics analyses: Leveraging single-cell RNA sequencing, epigenomics, and proteomics to dissect NSUN2-mediated regulatory networks and functional heterogeneity across different cell types and tumor microenvironments. Evaluating the reliability and applicability of NSUN2 as a diagnostic and prognostic biomarker to guide personalized therapeutic strategies. Designing small-molecule inhibitors or other intervention strategies targeting NSUN2, and exploring their combination with immune checkpoint inhibitors or other anti-tumor immunotherapies to enhance efficacy and overcome therapy resistance. In summary, a comprehensive understanding of NSUN2’s molecular mechanisms and clinical potential will provide novel theoretical insights and strategies for cancer treatment. Additionally, these studies will open new avenues for the application of RNA epigenetics in tumor immunity research.
